# Small-scale (sub-organ and cellular level) alpha-particle dosimetry methods using an iQID digital autoradiography imaging system

**DOI:** 10.1038/s41598-022-22664-5

**Published:** 2022-10-26

**Authors:** Robin Peter, Brenda M. Sandmaier, Michael P. Dion, Sofia H. L. Frost, Erlinda B. Santos, Aimee Kenoyer, Donald K. Hamlin, D. Scott Wilbur, Robert D. Stewart, Darrell R. Fisher, Kai Vetter, Youngho Seo, Brian W. Miller

**Affiliations:** 1 Department of Nuclear Engineering, University of California, Berkeley, CA USA; 2 Department of Radiology and Biomedical Imaging, University of California, San Francisco, CA USA; 3 Fred Hutchinson Cancer Center, Seattle, WA USA; 4 Division of Medical Oncology, Department of Medicine, University of Washington, Seattle, WA USA; 5 Oak Ridge National Laboratory, Oak Ridge, TN USA; 6 Department of Radiation Oncology, University of Washington, Seattle, WA USA; 7 Versant Medical Physics and Radiation Safety, Richland, WA USA; 8 Department of Radiation Oncology, Department of Medical Imaging, College of Medicine, University of Arizona, Tucson, AZ USA

**Keywords:** Targeted therapies, Medical research, Imaging techniques, Cancer therapy, Radiotherapy

## Abstract

Targeted radiopharmaceutical therapy with alpha-particle emitters (αRPT) is advantageous in cancer treatment because the short range and high local energy deposition of alpha particles enable precise radiation delivery and efficient tumor cell killing. However, these properties create sub-organ dose deposition effects that are not easily characterized by direct gamma-ray imaging (PET or SPECT). We present a computational procedure to determine the spatial distribution of absorbed dose from alpha-emitting radionuclides in tissues using digital autoradiography activity images from an ionizing-radiation quantum imaging detector (iQID). Data from ^211^At-radioimmunotherapy studies for allogeneic hematopoietic cell transplantation in a canine model were used to develop these methods. Nine healthy canines were treated with 16.9–30.9 MBq ^211^At/mg monoclonal antibodies (mAb). Lymph node biopsies from early (2–5 h) and late (19–20 h) time points (16 total) were obtained, with 10–20 consecutive 12-µm cryosections extracted from each and imaged with an iQID device. iQID spatial activity images were registered within a 3D volume for dose-point-kernel convolution, producing dose-rate maps. The accumulated absorbed doses for high- and low-rate regions were 9 ± 4 Gy and 1.2 ± 0.8 Gy from separate dose-rate curves, respectively. We further assess uptake uniformity, co-registration with histological pathology, and requisite slice numbers to improve microscale characterization of absorbed dose inhomogeneities in αRPT.

## Introduction

Despite advances in cancer treatment in recent decades, patients with refractory or metastatic cancers continue to face challenging outcomes after treatment with chemotherapy, radiotherapy, and biologic therapies^1^. Radiopharmaceutical therapy with alpha-particle emitters (αRPT) exploits the short range (10–100 µm) and high local energy deposition (up to 200 keV/µm linear energy transfer or LET) of alpha particles to therapeutic advantages. When an alpha-particle-emitting radioisotope is attached to a vector capable of targeting specific antigens on tumor cells, radiation can be efficiently delivered to diseased tissue with minimal dose to adjacent healthy cells. Trials have demonstrated significant therapeutic benefits in patients and resulted in U.S. Food and Drug Administration (FDA) approval of alpha-emitting ^223^Ra dichloride (Xofigo) for prostate cancer^2–4^.

The field of RPT is increasingly investigating quantitative imaging and dosimetry of patient-, tumor- and organ-specific radionuclide distributions to take full advantage of the modality’s potential for patient-informed prescriptions^5^. Sub-organ spatial dose characterization is particularly important to understand the effects of αRPT since therapeutic alpha particles (~ 2–10 MeV) only travel a few cell diameters in tissue, and standardized small-scale dosimetry methods have been identified as an unmet prerequisite of clinical translation^6,7^. Adaptations of gamma-ray imaging technologies to αRPT verification are challenging due to the lack of or minimal signal from long-range photons. In vivo assessment of alpha-emitters in tissues must contend with the extremely low administered activities of these procedures, which pose challenges to image quality^8,9^. Techniques using surrogate tracers introduce a systematic uncertainty in the radiopharmaceutical kinetics that may dominate the sub-organ effects being examined^6,8^.

Without robust in vivo imaging methods, sub-organ alpha-particle effects may be understood through ex vivo biodistribution and dosimetry studies using autoradiography^8,10^. Several bioimaging autoradiographs characterized by high resolution and large dynamic range—to extents currently unattainable with in vivo methods—have been developed for preclinical and clinical radiopharmaceutical characterization, including the α-camera^11^, alpha camera^12^, Timepix detector^13^, and iQID (ionizing-radiation quantum imaging detector) camera^14^. The iQID offers near-cellular resolution (~ 20 µm full-width at half maximum or FWHM), high detection efficiency (98% ± 1%), and real-time event-by-event quantitative imaging, and therefore has been used to obtain quantitative activity maps in αRPT studies^15–21^. However, few studies have derived quantitative absorbed dose maps at relevant spatial scales from autoradiographs because of the significant image processing requirements. Dosimetry using the α-camera has been done for a limited number of studies^15,22^, but the methods have not been fully articulated. We seek to codify a robust digital autoradiography dosimetry framework that can efficiently process many tissue sections for extensive αRPT studies.

In this work, we present methods for radiation dosimetry in alpha-particle pharmacokinetic studies using an iQID camera. Rapid imaging is accompanied by a suite of semi-automated Python scripts for quantitative activity estimation, image registration, absorbed dose and dose-rate estimation, analysis of uptake uniformity, and co-registration with hematoxylin and eosin (H&E) stains. Data from sixteen canine lymph nodes following administration of ^211^At-labeled anti-CD45 monoclonal antibody (mAb) for allogeneic hematopoietic cell transplantation conditioning is evaluated for demonstration and validation of the scripts. We show that mean dose metrics do not accurately characterize dose distribution, and therefore therapeutic effects, in tissues with inhomogeneous target expression. To improve data collection throughput, we assess the quantitative accuracy of using 1–3 tissue slices to evaluate dose rate, instead of 10–20 slices.

We follow the RPT nomenclature convention used elsewhere^5^, in which the term *dose* describes the radiation dose (ie, absorbed dose, in J/kg or Gy) rather than the mass quantity of pharmaceutical (as is used in non-radioactive therapies). This physical quantity is distinguishable from the administered activity, which is discussed in radioactive disintegrations per unit time (Bq or Ci) where applicable.

## Methods

### Alpha-RIT imaging experiments

Astatine-211 (^211^At) anti-CD45 mAb radioimmunotherapy (RIT) conditioning is a promising substitute for external-beam total-body irradiation (TBI) in hematopoietic cell transplantation (HCT) preparative regimens for hematologic malignancies, including leukemias and non-Hodgkin’s lymphoma^15,23^. This work used data from ^211^At-RIT studies for HCT in a canine model to develop algorithms broadly applicable to tissue-section studies. Canine lymphoma is a practical and clinically relevant model used in therapy studies due to its similarities to human lymphoma in anatomic forms, clinical presentations, cellular surface markers, and therapeutic response^24,25^.

Data were collected as previously described^15^: nine healthy canines were treated with 16.9–30.9 MBq ^211^At/mg mAb (Supplemental Table S1). Lymph node biopsies were obtained at early (2–5 h) and late (19–20 h) time points (n = 16). Subjects weighed between 7.9–13 kg and received either 0.5 or 0.75 mg/kg of ^211^At-labelled anti-CD45 mAb CA12.10C12-B10 (8.44–23.2 MBq/kg injected activity, IA). iQID imaging and activity, dose-rate, and absorbed dose calculations proceeded as described below.

### Ethics approval

All procedures conducted were approved by the Fred Hutchinson Cancer Center Institutional Animal Care And Use Committee (IACUC)^26^. Fred Hutchinson is registered as a research facility with the USDA (91-R-0081), has a Letter of Assurance on file with PHS/OLAW (D16-00142), and is fully accredited by AAALAC International. The study design and methods reported previously^15^, combined with the reporting in the current manuscript, follow recommendations in the ARRIVE guidelines.

### Sequential sectioning

The ^211^At decay series contains two alpha-particle emissions from ^211^At and progeny radionuclide ^211^Po, with respective energies of 5.87 and 7.45 MeV that correspond to ranges in water of 48 and 70 µm^27^. 10–20 consecutive 12-µm cryosections that span the range and each allow alpha-particle escape were extracted from each biopsy to estimate the absorbed-dose-rate of the alpha particles in the central slice. Activity was measured simultaneously for each slice in the series using an iQID device. We collected data for as long as possible before the next biopsy to reduce statistical uncertainty and utilize available imaging time (about 15 h, or twice the 7.2-h half-life of ^211^At). We discarded samples with major tears or folds from post-processing analysis and replaced them with duplicates of neighboring slices. Two full biopsies from one canine were not analyzed because the discarded samples outnumbered the acceptable ones. Challenges associated with sample collection are discussed further in the Slice Minimization section.

### iQID camera

**Figure 1:**
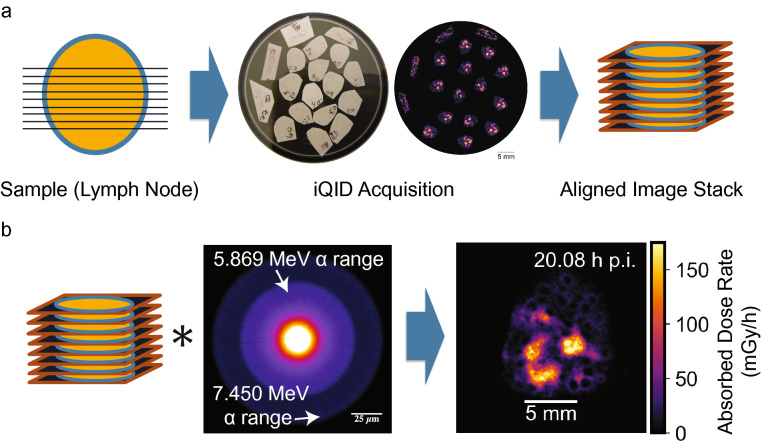
Overview of dosimetry with iQID digital autoradiography. (**a**) Cartoon schematic of data acquisition. Thin (12 µm) cryosections were cut from a tissue biopsy, imaged in 2D with an iQID device, and re-aligned into a 3D volume using image processing. (**b**) Convolution of the registered activity image stack with a 3D ^211^At Monte Carlo dose kernel produced dose-rate maps of canine lymph node slices. iQID scale bars (5 mm) were based on field-of-view reference images and voxel sizes.

The iQID operational mechanism, scintillator options and characteristics, device schematic, and event readout systems are described in detail in previous work^14,28^. The same set-up is used in these experiments. In brief, the device comprises a scintillator in direct contact with a microchannel plate image intensifier and lens that projects scintillation light onto a 4 megapixel (2048 × 2048) Point Grey Research Grasshopper® 3 camera with a CMOSIS CMV4000 CMOS sensor^28^. Alpha particles that escape the thinly sliced tissue deposit in the ZnS:Ag scintillation screen (EJ-440; Eljen Technology), which is chosen for its commercial availability and extremely high light yield (~ 95,000 photons/MeV)^29^. The scintillation light is amplified with a spatial-information-preserving image intensifier and imaged onto the CMOS sensor using a CCTV camera lens. Each alpha-particle interaction event appears in an image frame as a small cluster of contiguous pixels on the CMOS camera. Multiple alpha-particle events may be present in a single image frame and are processed using centroid estimation into single events in listmode data with spatial and temporal information. iQID is thus an event-by-event measurement device in which one count is recorded per detected alpha decay. The efficiency of iQID for alpha particles between 3.70 and 7.95 MeV using ZnS:Ag was measured previously to be 98% ± 1% in a 4π geometry^14^, or one-half that for the 2π geometry used in this experiment. Images were measured at 20–30 frames per second (33–50 ms exposure time). A calibration scale determined the effective pixel size for a measurement (10–30 µm). Prior work^14^ has characterized the intrinsic detector resolution up to 20 µm (FWHM). Real-time iQID images and acquisition settings are viewed in a custom-developed LabVIEW™ acquisition software. A top-down view of tissue samples on the iQID device, and the subsequent iQID event image, can be seen in Fig. 1a (center).

### Image registration

**Figure 2:**
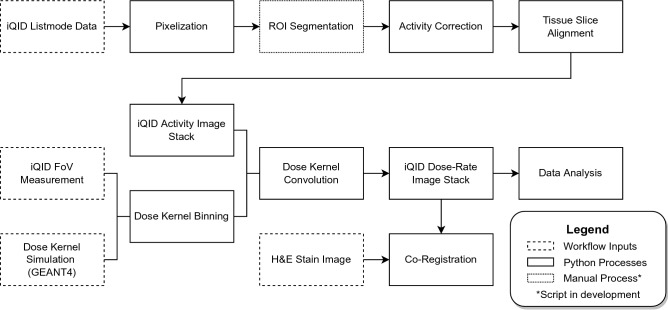
Detailed image processing flowchart showing sequence of automated and semi-automated image processing steps.

We processed iQID autoradiographs into dose-rate images using a combination of open-source Python libraries, including scikit-image (0.18.1)^30^, OpenCV (4.0.1)^31^, and PyStackReg (0.2.5)^32,33^. Figure 1 overviews the concept of dosimetry with digital autoradiography, and Fig. 2 details the sequence of processing steps required. Scripts for these methods are available as an in-development Python package on Github at https://github.com/robin-peter/iqid-alphas (https://doi.org/10.5281/zenodo.7117835).

Multi-slice autoradiographs were segmented into regions of interest (ROIs) corresponding to discrete tissue slices. Detected events in each ROI underwent temporal binning and exponential correction for ^211^At decay by a least-squares residual optimization exponential fit to the temporal histogram of counts over the course of imaging. This process yielded a spatial activity snapshot of each tissue slice at the time of biopsy.

We registered activity images of consecutive slices within a 3D volume by approximating each thin slice (12 µm) as a minimally distorted 2D transformation of its neighbor (Fig. 1a). The orientation was roughly obtained by minimizing the mean-squared error (MSE) between image intensities through rotation angles. Packaged rigid-body transformations (translation and rotation) were then applied for precise alignment (PyStackReg). Small errors in the cumulative activity introduced by rotation were recorded and corrected for with a scalar compensation factor (2.2% mean error).

### Dose rate estimation

**Figure 3:**
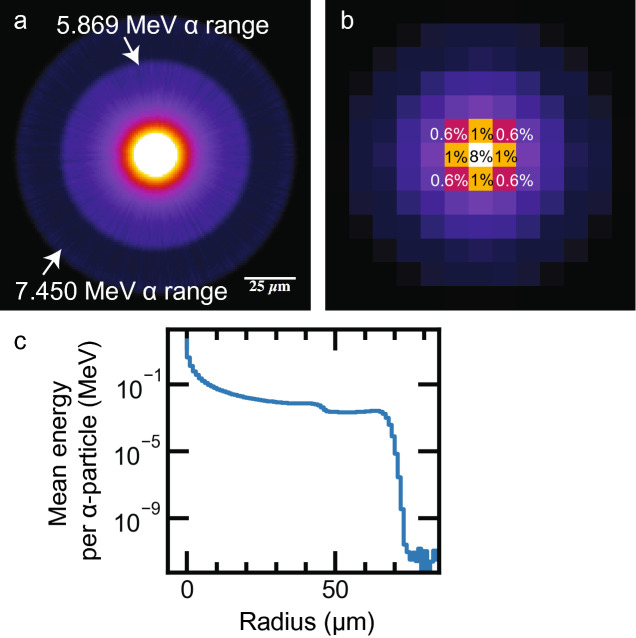
Monte Carlo energy deposition kernel for ^211^At alpha-particles in water [10^7^ events, GEANT4 ^36^]. (**a**) 1-µm voxel kernel (151 × 151 × 151 µm) showing two alpha-decay pathways for ^211^At. (**b**) Kernel with percentage energy deposition in voxels binned to 12 µm. Energy deposition falls rapidly beyond adjacent 12-µm tissue slices. (**c**) Semi-log radial profile of (**a**) normalized to one alpha-particle, showing fall-off with distance.

We used a dose-point-kernel (DPK) convolution method with a Monte Carlo (MC) kernel to estimate spatial dose rates in tissues. DPK convolution has been investigated with analytic and MC-generated kernels, especially for internal dosimetry of gamma- and beta-emitters^6,22,34,35^. We generated the 3D energy deposition kernel (Fig. 3) with 1-µm voxels using the GEANT4 Monte Carlo framework^36^, simulating 10^7^ alpha-particle emissions from the ^211^At decay chain in water. ^211^At and ^211^Po were assumed to be in secular equilibrium, and only the alpha particles were generated as primaries. The kernel was averaged radially and binned to the voxel size of the iQID image stack (XY: 10–30 µm; Z: 12 µm). The mean statistical uncertainty associated with any non-zero 1-µm voxel was 1.1% after radial averaging and before image-specific binning.

Convolution of the 3D activity image stack $A\left( r \right)$ with the energy deposition kernel $K\left( r \right)$, and inclusion of mass through voxel density $\rho \left( r \right)$ and volume $V\left( r \right)$, produced a 3D instantaneous dose-rate snapshot $\dot{D}\left( r \right)$ at the time of biopsy (Fig. 1b):

$$\dot{D}\left( r \right) = \int \frac{{K\left( {\left| {r^{\prime} - r} \right|} \right)}}{\rho \left( r \right)V\left( r \right)}A\left( {r^{\prime}} \right)d^{3} r^{\prime}.$$

Here, we have used the nomenclature of ICRU Report 96^6^, where $r$ represents the source position and $r^{\prime}$ represents the target position. The (discrete) convolution integral was calculated using a Fast Fourier Transform (FFT)-based packaged Python method. We used data from the central slice of the image stack for analysis because accurate calculation may require measured data spanning the full ^211^At alpha-particle range on both sides of a slice, which is not guaranteed for peripheral slices.

### Histological fusion

Tissue slices consecutive with each sample series were stained with hematoxylin and eosin (H&E) using a standard protocol. We digitally co-registered H&E images with iQID dose-rate maps to compare pathological information and agent uptake. Our approach was to down-sample the high-resolution H&E images (50–200 MB) by a factor of 20 to compute the appropriate transformation for the iQID images, then map the transformed iQID images onto the full-resolution H&E stains.

We first cast all images to binary for alignment. Rough thresholds for the down-sampled H&E images were obtained using Otsu's method (scikit-image)^37^, a variance-based procedure that separates an image by clustering the intensity histogram. A smaller threshold (one-tenth of an Otsu threshold) was used for the sparser iQID images to obtain binaries covering the whole spatial extent of the lymph node. We obtained pixel sizes from scale bars (H&E) and field-of-view measurements (iQID), then scaled the iQID images by the corresponding factor using scikit-image's rescale function. The same-dimension images were co-registered using the described methods for slice alignment, including MSE intensity comparison and 2D transformations. Since H&E slices were spatially separated from iQID-measured central slices by 50–100 µm, we permitted some misalignment and shear transformations during co-registration. Threshold detection, scaling factors, and 2D transformations were all calculated and applied automatically, with manual corrections for reflections (slices face-up versus face-down).

### Slice minimization

Dosimetry studies that investigate multiple time points and tissue types can be bottlenecked by the requisite preparation of 10–20 12-µm tissue slices. Collection of a sequential sample series without tears or folds requires considerable skill and may limit the accessibility of our dosimetry methods. Additionally, the device field of view (40- and 115-mm diameter iQID configurations were used) can limit imaging throughput. Imaging throughput is important for experiments using short-lived radionuclides, in which several tissues from the same biopsy or sacrifice time should be imaged before extensive signal decay, and long-lived radionuclides, in which imaging times are long to obtain statistically significant counts. Anticipating these throughput challenges, we examined whether dose-rate estimates may be obtained using fewer slices using simulated variations of the DPK convolution procedure.

We hypothesized that our dosimetry routine would yield similar results whether a few surrounding slices (1–3 total) or a full set (up to 10 on either side of the center slice) were used to calculate the central-slice dose-rate map. Figure 3 illustrates our reasoning, showing the average per-alpha-particle energy deposition kernel binned to the cryosection slice thickness (12 µm) (Fig. 3a), energy distribution in selected voxels (Fig. 3b), and the kernel’s radial profile, which falls off rapidly with distance (Fig. 3c). The initial fall-off of the kernel is attributable to the dispersion of alpha-particle flux in the 3D medium, resulting in a $1/r^{2}$ decrease in energy deposition, with the two ^211^At alpha-particle Bragg peaks seen on top. The sharp cut-off at 70 µm indicates that minimal energy is deposited beyond the 7.45-MeV ^211^Po alpha-particle range. Therefore, decay events more than six slices away (72 µm) would not contribute significant dose to the measured slice, and slices up to that point contribute diminishing dose compared to those near the center.

### Slice contribution method

We tested our hypothesis by simulating limited-data trials in which only one or several consecutive slices were acquired from the center. These spatial dose-rate calculations are identical to those described above, except that only *N* = *1, 3, 5, … N*_*max*_ central slices are used in the 3D iQID activity image stack, whereas *N*_*max*_ slices were used in the original analysis. The summed dose rate of all voxels in the central slice, $\dot{D}_{N}$, was recorded for comparison to the dose rate using all available slices, $\dot{D}_{0}$. To preserve symmetry in the central slice measurement, we simulated trials at odd slice numbers by adding one additional slice on each side to increment *N*.

To obtain accurate dose-rates using this method, a correction factor to scale the limited-slice data was obtained by empirically fitting $\dot{D}_{N} /\dot{D}_{0}$ with respect to *N*. However, determination of the correction factor required a full set (10–20 slices) of data. Although this method could reduce the slice requirements of subsequent procedures using the same data set, we sought an alternative method without this constraint.

### Cloning method

We investigated an alternative limited-data method without the initial slice requirements in which the unmeasured tissue in a limited-slice simulation was digitally replaced by duplicates, or clones, of the outermost measured slices. For a limited-data trial of *N* = *1, 3, 5, … N*_*max*_ central slices, the remaining *N*_*max*_*-1, N*_*max*_*-3, N*_*max*_*-5, … 0* slices were duplicates of the external slices on either side. DPK convolution then proceeded as described above. Cartoon schematics of the two methods are shown in Fig. 7a.

## Results

### Uptake non-uniformity

**Figure 4:**
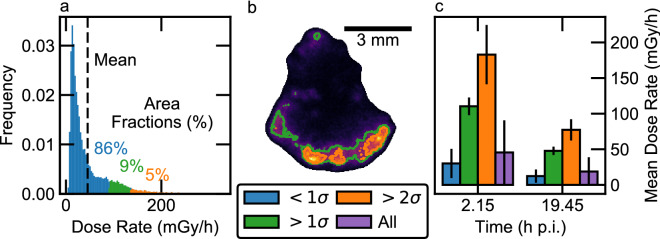
Uniformity analysis of instantaneous dose-rates at biopsy. (**a**) Dose-rate-area histogram of a slice of canine lymph node with regions segmented by deviation from the mean (dashed line). (**b**) Corresponding regions highlighted on the dose rate image in same colors. (**c**) Dose rates from two biopsies at different time points from the same canine. A mean value assessed over the whole organ underestimated the dose imparted to certain regions of tissue (5–6% of area) by a factor of three.

Our studies confirmed localized high-activity regions in a background of low-activity tissue. Tissues were digitally segmented from non-tissue background with an automatically generated contour mask. Voxel values of instantaneous dose rate at biopsy were binned into a dose-rate-area histogram (Fig. 4a), which we partitioned into three regions defined by standard deviations from the mean. Areas corresponding to these regions were highlighted on the corresponding dose-rate image (Fig. 4b).

In a two-biopsy study from one representative canine, the respective mean dose rates were 46 ± 45 mGy/h (at 2.15 h p.i.) and 19 ± 20 mGy/h (at 19.45 h p.i.). The uncertainties, which represent one standard deviation (σ), reflect the long high-dose tail of the dose-rate-area histogram (Fig. 4a). Therefore, 14% and 13% of the tissue area (> 1σ from the mean) showed dose-rate values over double the whole-organ mean in the respective biopsies, and 5% and 6% of the tissue area (> 2σ) exceeded three times the whole-organ mean. Figure 4c shows these discrepancies, with mean dose rates in each sub-region (< 1σ, > 1σ, > 2σ, and over the whole lymph node) in the two biopsies from early and late time points.

### Absorbed dose estimation

**Figure 5:**
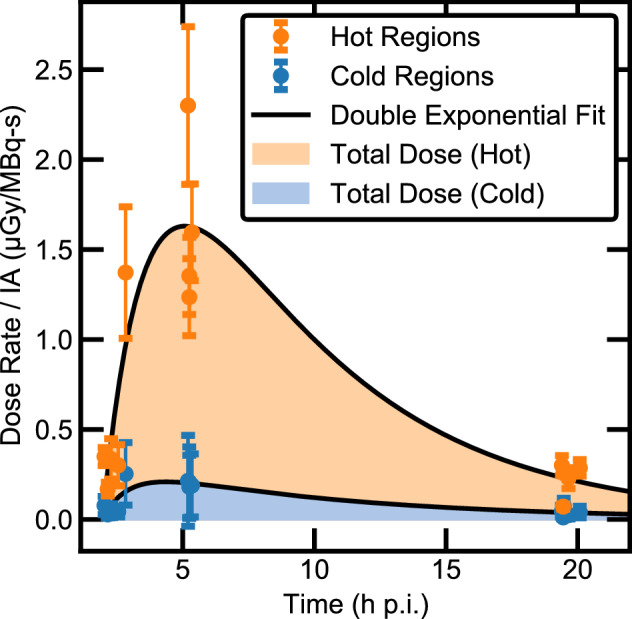
Dose rate curves separated into high-dose-rate (orange, “hot”) and low-dose-rate (blue, “cold”) regions. Double-exponential fits ($y = ae^{{ - b\left( {x - m} \right)}} - ce^{{ - d\left( {x - n} \right)}} , \chi^{2} /\nu = 19.5, 2.2$ for hot and cold, respectively) use data from 14 biopsies across eight canine models, normalized to the injected activity of each study (8.44–23.2 MBq/kg). Shaded regions show curve-integration to estimate total absorbed dose, yielding hot-region doses of 9 ± 4 Gy and cold-region doses of 1.2 ± 0.8 Gy.

Dose rates were estimated with a batch script for 16 biopsies at early and late time points with two biopsies discarded for sample flaws (see Methods; n = 14). We assumed that uptake and decay kinematics were similar between canine subjects to compare IA-normalized dose rates across studies. Since uniformity analysis suggested that the mean dose rate may not be a representative metric for all regions in a sample, the measurement data was separated into high- (> 2σ, “hot”) and low- (< 1σ, “cold”) dose-rate regions of each lymph node section. The mean dose rate within each subset is plotted with time in Fig. 5.

To estimate dose, the data were fit to a double exponential function using least-square residual optimization (Fig. 5). Standard deviations were calculated within each data subset for each biopsy and used as error to obtain reduced chi-squared values of 19.5 and 2.2 for the hot and cold regions. These quality-of-fit values deviate from unity more than typically expected for quantitative analysis, particularly for the high-activity regions. However, this result is not surprising given the limited sample points (n = 14 for a six-parameter fit) and variability within the data set. The study was not controlled to address specific regions within lymph nodes, resulting in samples with a variety of cuts (coronal, sagittal, transverse) and volumes (half-node, whole node). Segmentation of the regions by dose rate may therefore result in comparisons between different lymph node structural regions with high uptake.

Integration of the hot and cold dose-rate curves to six ^211^At half-lives yielded upper and lower bounds for the absorbed dose received in the lymph node tissue slice. Uncertainties were roughly estimated by calculating the dose from a hypothetical dose-rate curve scaled to pass through the maximum value in the data set. For a mean IA of 150 ± 60 MBq (4.1 ± 1.6 mCi), the doses received in hot and cold regions were found to be 9 ± 4 Gy and 1.2 ± 0.8 Gy, respectively.

### Histological fusion

**Figure 6:**
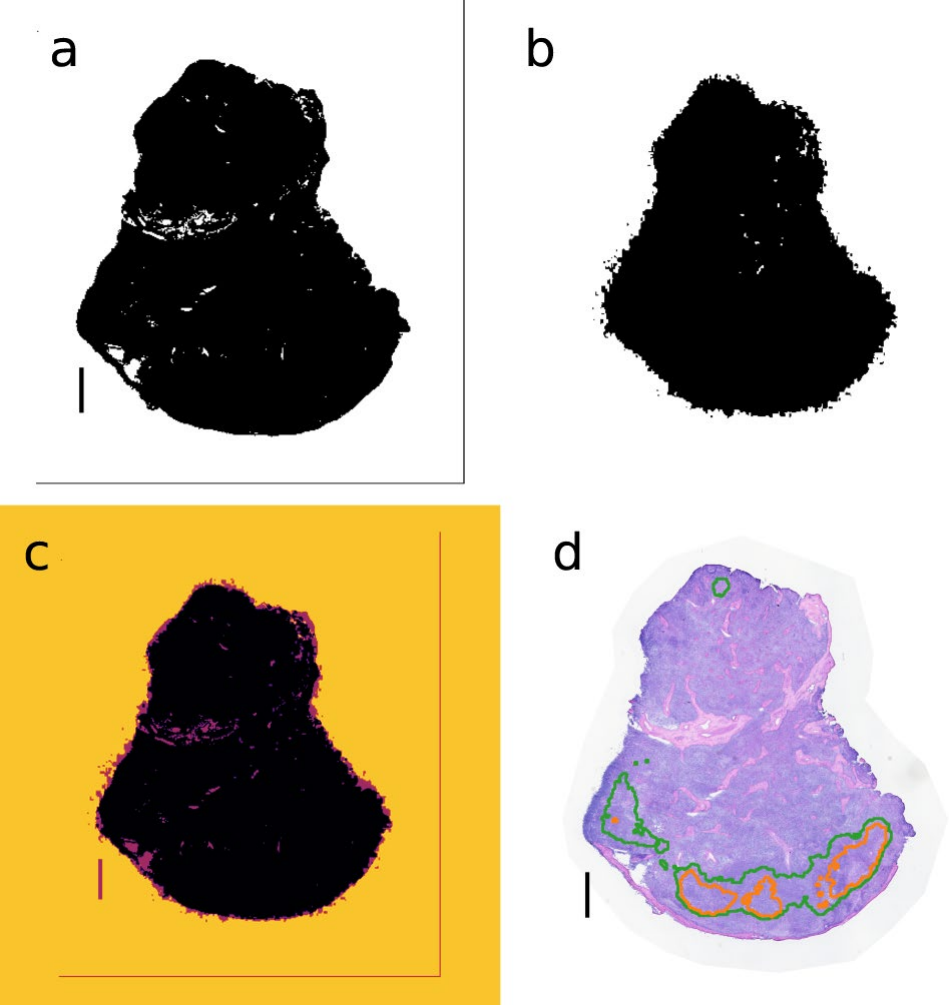
Registration procedure between iQID dose-rate images and H&E-stained images. (**a**) H&E thresholded binary image. (**b**) iQID dose-rate-map thresholded binary image. (**c**) Co-registration of (**a**) and (**b**) using scale factors and 2D transformations. (**d**) Contours of high-activity regions from Fig. 4b overlaid on H&E image. Scale bars show 1 mm.

Figure 6 shows the registration procedure for iQID dose-rate images and the H&E pathology images described in the Methods section. Contours of high-dose-rate regions were overlaid on the H&E image for pathological comparison (Fig. 6d).

### Slice minimization

**Figure 7:**
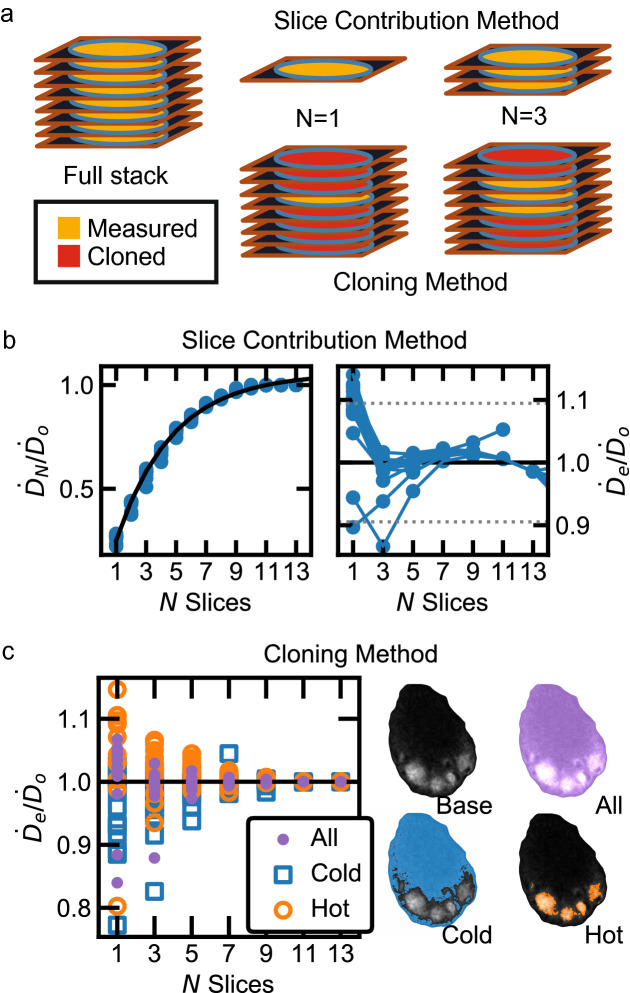
Slice minimization analysis. (**a**) Cartoon schematics showing slice contribution and cloning methods for approximating dose rates using limited data. (**b**) (Left) Cumulative contribution of *N* slices, $\dot{D}_{N}$, towards central-slice dose rate $\dot{D}_{0}$. An exponential fit (black line, *χ*^*2*^*/ν* = 1.73) provided a method to scale up a low-slice dose rate image. (Right) Evaluation of the slice contribution method by comparing $\dot{D}_{e}$ to $\dot{D}_{0}$. The mean deviation of $\dot{D}_{e}$ from $\dot{D}_{0}$ (14 biopsies) using once slice was 9.5% (gray dashed lines). (**c**) (Left) Evaluation of the cloning method in hot and cold data subsets. (Right) Mosaic of lymph node slices highlighted to show evaluated regions. The mean deviation of single-slice $\dot{D}_{e}$ from $\dot{D}_{0}$ (14 biopsies) was 4.8%, but no full-slice data were otherwise required. High density of hot data points above, and of cold data points below, the $\dot{D}_{e} /\dot{D}_{0} = 1$ line reflects overemphasis of features from the measured slice.

Figure 7a illustrates the two post-processing methods to estimate dose rate using limited numbers of tissue slices. In the slice contribution method, studies limited to *N* slices had slice contributions $\dot{D}_{N}$, shown as fractions of the all-slices (control study) total dose rate $\dot{D}_{0}$ (Fig. 7b, left). An exponential fit through this data (*χ*^*2*^*/ν* = *1.73*) provided an empirical scalar correction factor $C_{N}$ to yield a corrected dose-rate estimate $\dot{D}_{e} = C_{N} \dot{D}_{N} .$ The efficacy of the fit-scaling procedure was evaluated (Fig. 7b, right) by comparing $\dot{D}_{e}$ to $\dot{D}_{0}$. Loss of accuracy past *N* = *11* (130 µm) reflects that an asymptotic model was used to fit data that reaches unity at sufficient *N*. Across 14 biopsies from eight canine models, the mean deviation of $\dot{D}_{e}$ from $\dot{D}_{0}$ was 9.5% using one slice and 2.4% using three slices.

The slice contribution method required a full control study (10–20 slices) to calculate alpha-particle dose rates. We proposed an alternative “cloning” post-processing method in which the external slices of a limited-slice trial were duplicated outwards to span the alpha-particle range before DPK convolution.

Across 14 biopsies from eight canine models, the mean deviation of single-slice $\dot{D}_{e}$ from $\dot{D}_{0}$ using the cloning method was 4.8% using one slice and 1.8% using three slices. The cloning method attempts to obtain quantitatively accurate dose rates without having the spatial information of multiple slices and assumes that surrounding tissue contains identical features in the same spatial locations. We tested this assumption by assessing the same dose-rate comparison metric within each data subset (hot regions, cold regions, and whole lymph node means: Fig. 7c). The mean deviations of $\dot{D}_{e}$ from $\dot{D}_{0}$ for hot and cold regions, respectively, were 7.9%/3.9% and 6.5%/4.3% (single/triple slices). This bias, which decreases with *N*, demonstrates dose-rate overestimation (above the $\dot{D}_{e} /\dot{D}_{0} = 1$ line) in hot regions and underestimation (below the $\dot{D}_{e} /\dot{D}_{0} = 1$ line) in cold regions.

## Discussion

This work adds a suite of quantitative methods for small-scale dosimetry to the digital autoradiography tools developed by Bäck, Jacobsen, and Miller^11,14,22^. Digital autoradiographs have been used to quantify activity distributions in preclinical αRPT studies, but with a few exceptions, absorbed dose estimates in αRPT pharmacokinetic studies are obtained from biodistribution studies that are not spatially sensitive within organs^17–21^.

Our work constitutes an improvement to previous digital autoradiography-based αRPT dosimetry^15,22^ in three ways:

- The computational and image processing methods, including ROI segmentation, activity correction, slice registration, and MC DPK convolution, have not been fully described in previous works to our knowledge;
- The iQID camera is a self-contained system that measures activity directly (event-by-event) rather than using a secondary gamma-counting measurement, as with the α-camera. Therefore, the uncertainty associated with dosimetry measurements is reduced;
- We have introduced elements of automation to improve efficiency of data collection and processing, incorporated histogram segmentation for assessment of non-uniformity, and proposed methods to reduce prohibitive slice requirements of these studies. Scripts are available as an in-development Python package on Github at https://github.com/robin-peter/iqid-alphas (https://doi.org/10.5281/zenodo.7117835).

Our procedure does not trace individual alpha-particle tracks or their stochastic effects in specific cells but rather calculates absorbed dose with a mean Monte Carlo (MC) energy deposition kernel. We thus classify our approach as small-scale (sub-organ and cellular level) rather than a true stochastic microdosimetric method, using the titular convention^21^. Use of the DPK kernel allows for extension of this method to other radionuclides^38^ or non-water-like tissues such as bone and lungs^39^ by generating a new kernel. The authors are currently conducting studies of therapeutically conjugated ^225^Ac and ^227^Th in a murine model with these methods.

Direct MC, DPK convolution, and MIRD S-coefficients (or S-values) are the three current dosimetry methodologies identified by ICRU Report 96 as relevant for calculation of absorbed dose in RPT^6^. The S-coefficient method is often regarded as the most practical and closest to a clinical standard, but it usually assumes uniform distribution of dose within an organ, which is often inaccurate in αRPT. Our procedure uses MC and DPK convolution in a procedural workflow, mostly contained in one non-proprietary software (Python), that can be replicated to obtain dosimetry results in systems of other radionuclides and tissues by tuning only a few specific components. Future work should compare DPK methods using iQID with actively researched S-coefficient approaches that model dose inhomogeneity, such as voxel-level or cellular-scale S-values (ie MIRDcell simulation)^6,40,41^. However, voxel-level S-values are typically assessed at scales much larger than iQID (PET and SPECT resolutions, > 0.5 mm), and MIRDcell simulation results may be challenging to map to the non-cellular iQID device. The comparison between DPK and micro-scale S-value methods for iQID is therefore a non-trivial task.

Alpha-particle dose varies widely at the cellular level because the short particle range results in dose localized around expression of the target antigen. We confirmed that exclusively reporting the mean dose, as is common in biodistribution studies, may insufficiently characterize therapeutic effects in sub-organ tissues with inhomogeneous radiopharmaceutical uptake. Research that investigates the relationship between this microscale dose nonuniformity and biologic effect has been identified as a key avenue for progress in αRPT^6^. Our method of digital dose segmentation and co-registration with pathological information allows dose estimation on the scale of nonuniformities and promotes co-investigation of biologic effects. The methods presented in this work can also be generalized to report the RBE-weighted dose distribution for endpoints such as DNA double strand break induction^42^ and reproductive cell survival^43^ to further assess the clinical impact of αRPT.

We characterized two post-processing methods to significantly reduce the number of cryosections needed, using only 1–3 12-µm slices to obtain dose rate estimates in the center slice instead of using 10–20 slices for that data point. Both methods showed similar convergence at higher slice numbers*,* but the cloning method had slightly superior single-slice accuracy in our trials (4.8% vs 9.5%)*.* The cloning method does not require a full data set to develop an empirical correction factor—unlike the slice contribution method—and thus could be applied to limited-data studies without prior experience. The main limitation of the cloning method is the assumption that the spatial distribution of radioactive hotspots does not vary significantly with tissue depth, which results in a systematic overemphasis of features in the measured slice. Differences in morphological architecture and physiology from one section to the next will thus affect the accuracy of the cloning method. The observed difference was small for the canine lymph node data used, but other tissue types may exhibit greater structural inhomogeneity. Anatomically diverse tissues may require a full range of slices for accurate dose calculations using the slice contribution or full-sequence methods instead. However, all DPK methods are ill posed to measure samples with small-scale, heterogeneous material structures (such as bone marrow trabeculae) because the interfaces between tissue and non-tissue (e.g., air or bone) would be inaccurately reflected unless specifically simulated in the DPK.

Conversely, dosimetry calculations for a 12-µm slice may not characterize the rest of the biopsy beyond the alpha-particle range. Multiple sparse samples are likely needed to assess the 3D dose distribution in a whole organ. Cryosections should be cut at a consistent location to minimize geometric effects (e.g., increased uptake in outer tissue layers) or structural effects (e.g., increased presence of cortical follicles along a longitudinal compared to a transverse plane). Future work assessing the variation of samples within an organ would be valuable for improving the accuracy and consistency of dose estimates.

In informal terms, the methods proposed here should be considered a suggested recipe, rather than a fixed prescription, for αRPT dosimetry using digital autoradiography. Follow-up studies should aim to optimize the components, including the registration procedure, microdosimetry model (DPK or otherwise), and experimental parameters. The dose contribution of the ^211^At electron-capture branch was not quantified in the dose kernel and should be investigated in future work. A simple algorithm was sufficient to align the canine lymph nodes in this study, but more robust procedures may be needed for complex tissues. Rigid 2D transformations were applied assuming that each slice did not distort significantly in 12 µm, which may not be the case with thicker slices. This assumption is untrue at distances as low as 50 µm away, as was observed in the registration of iQID images with H&E-stained slices. Thicker slices also degrade depth resolution and detection efficiency as fewer alpha particles escape the tissue, but they are easier to cut from biopsies without sample handling errors. Quantifying the trade-offs between spatial resolution and precise dose localization, and between slice thickness and detection efficiency, would provide insight into the optimal field-of-view and cryotome setups.

## Conclusion

This work constitutes a set of methods to obtain quantitative, spatially sensitive ex vivo alpha-particle absorbed dose measurements, which are needed for the clinical translation of alpha-particle therapy. We present advanced tools, automated where possible, to quantify cellular-scale absorbed dose and dose rates with single-particle digital autoradiography imaging and assess uptake uniformity, histological pathology, and requisite slice numbers. These methods improve the feasibility of preclinical and clinical biopsy absorbed dose measurement to further our understanding of alpha-particle radiopharmaceuticals and their biologic effects at the cellular level.

## Supplementary Information

Supplementary Information.

## Data Availability

The datasets analyzed during the current study are available from the corresponding author on reasonable request.

## References

[1] Sgouros G, Bodei L, McDevitt MR, Nedrow JR (2020). Radiopharmaceutical therapy in cancer: Clinical advances and challenges. Nat. Rev. Drug Discov..

[2] Makvandi M (2018). Alpha-emitters and targeted alpha therapy in oncology: From basic science to clinical investigations. Target. Oncol..

[3] Kratochwil C (2016). ^225^Ac-PSMA-617 for PSMA-targeted α-radiation therapy of metastatic castration-resistant prostate cancer. J. Nucl. Med..

[4] Frantellizzi V (2020). Targeted alpha therapy with thorium-227. Cancer Biother. Radiopharm..

[5] Lawhn-Heath C (2022). Dosimetry in radionuclide therapy: The clinical role of measuring radiation dose. Lancet Oncol..

[6] Sgouros G (2021). ICRU REPORT 96, dosimetry-guided radiopharmaceutical therapy. J. ICRU.

[7] Tronchin S, Forster JC, Hickson K, Bezak E (2022). Dosimetry in targeted alpha therapy. a systematic review: Current findings and what is needed. Phys. Med. Biol..

[8] Seo Y (2019). Quantitative imaging of alpha-emitting therapeutic radiopharmaceuticals. Nucl. Med. Mol. Imaging.

[9] Hindorf C, Chittenden S, Aksnes A-K, Parker C, Flux GD (2012). Quantitative imaging of ^223^Ra-chloride (Alpharadin) for targeted alpha-emitting radionuclide therapy of bone metastases. Nucl. Med. Commun..

[10] Sgouros G (2010). MIRD pamphlet no. 22 (Abridged): Radiobiology and dosimetry of α-particle emitters for targeted radionuclide therapy. J. Nucl. Med..

[11] Bäck T, Jacobsson L (2010). The α-camera: A quantitative digital autoradiography technique using a charge-coupled device for Ex Vivo high-resolution bioimaging of α-particles. J. Nucl. Med..

[12] Yamamoto S, Hirano Y, Kamada K, Yoshikawa A (2020). Development of an ultrahigh-resolution radiation real-time imaging system to observe trajectory of alpha particles in a scintillator. Radiat. Meas..

[13] Darwish RA, Staudacher AH, Bezak E, Brown MP (2015). Autoradiography imaging in targeted alpha therapy with timepix detector. Comput. Math. Methods Med..

[14] Miller BW (2015). Quantitative single-particle digital autoradiography with α-particle emitters for targeted radionuclide therapy using the iQID camera. Med. Phys..

[15] Frost SHL (2015). α-Imaging confirmed efficient targeting of CD45-positive cells after ^211^at-radioimmunotherapy for hematopoietic cell transplantation. J. Nucl. Med..

[16] Lamart S (2017). Actinide bioimaging in tissues: Comparison of emulsion and solid track autoradiography techniques with the iQID camera. PLoS ONE.

[17] Miller, C. In *European Association of Nuclear Medicine 34th Annual Congress* (Virtual, 2021)

[18] Vargas CS (2020). Multi-regional dosimetry of mouse kidneys for beta-emitters. Eur. J. Nucl. Med. Mol. Imaging.

[19] Dekempeneer Y (2020). Therapeutic efficacy of ^213^Bi-labeled sdAbs in a preclinical model of ovarian cancer. Mol. Pharm..

[20] Tabatadze G, Miller BW, Tolmachev SY (2019). Mapping ^241^am spatial distribution within anatomical bone structures using digital autoradiography. Health Phys..

[21] Hobbs RF (2016). Application of small-scale (sub-organ and cellular level) alpha-particle specific dosimetry model in tumors and kidneys in a pre-clinical model of metastatic prostate cancer using ^211^At-YC-I-27, a PSMA-targeting ligand for metastatic prostate cancer. Brachytherapy.

[22] Bäck T (2014). Image-based small-scale 3D-dosimetry in targeted alpha therapy using voxel dose-point kernels and alpha camera imaging of serial tissue sections. J. Nucl. Med..

[23] Chen Y (2012). Durable donor engraftment after radioimmunotherapy using α-emitter astatine-211–labeled anti-CD45 antibody for conditioning in allogeneic hematopoietic cell transplantation. Blood.

[24] Ito D, Frantz AM, Modiano JF (2014). Canine lymphoma as a comparative model for human non-Hodgkin lymphoma: Recent progress and applications. Vet. Immunol. Immunopathol..

[25] Villarnovo D, McCleary-Wheeler AL, Richards KL (2017). Barking up the right tree: Advancing our understanding and treatment of lymphoma with a spontaneous canine model. Curr. Opin. Hematol..

[26] *Guide for the Care and Use of Laboratory Animals*. 8 edn, (National Academies Press (US), 2011). 21595115

[27] Berger, M. J. *et al. Stopping-Power & Range Tables for Electrons, Protons, and Helium Ions* (2009).

[28] Miller BW (2014). The iQID camera: An ionizing-radiation quantum imaging detector. Nucl. Instrum. Methods Phys. Res. A.

[29] Dorenbos P (2002). Light output and energy resolution of Ce3+−doped scintillators. Nucl. Instrum. Methods Phys. Res. A.

[30] Walt van der S (2014). scikit-image: Image processing in Python. PeerJ.

[31] Itseez. Open Source Computer Vision Library (2015).

[32] Lichtner, G. PyStackReg (2021).

[33] Thevenaz P, Ruttimann UE, Unser M (1998). A pyramid approach to subpixel registration based on intensity. IEEE Trans. Image Process.

[34] Giap HB, Macey DJ, Bayouth JE, Boyer AL (1995). Validation of a dose-point kernel convolution technique for internal dosimetry. Phys. Med. Biol..

[35] Peer-Firozjaei M, Tajik-Mansoury MA, Geramifar P, Parach AA, Zarifi S (2021). Implementation of dose point kernel (DPK) for dose optimization of ^177^Lu/^90^Y cocktail radionuclides in internal dosimetry. Appl. Radiat. Isot..

[36] Agostinelli S (2003). Geant4—a simulation toolkit. Nucl. Instrum. Methods Phys. Res. A.

[37] Otsu N (1979). A threshold selection method from gray-level histograms. IEEE Trans. Syst. Man Cybern..

[38] Graves SA, Flynn RT, Hyer DE (2019). Dose point kernels for 2174 radionuclides. Med. Phys..

[39] Khazaee Moghadam M, Kamali Asl A, Geramifar P, Zaidi H (2016). Evaluating the application of tissue-specific dose kernels instead of water dose kernels in internal dosimetry: A Monte Carlo study. Cancer Biother. Radiopharm..

[40] Vaziri B (2014). MIRD pamphlet no. 25: MIRDcell V2.0 software tool for dosimetric analysis of biologic response of multicellular populations. J. Nucl. Med..

[41] Katugampola S, Wang J, Prasad A, Sofou S, Howell RW (2022). Predicting response of micrometastases with MIRDcell V3: Proof of principle with 225Ac-DOTA encapsulating liposomes that produce different activity distributions in tumor spheroids. Eur. J. Nucl. Med. Mol. Imaging.

[42] Stewart RD (2015). Rapid MCNP simulation of DNA double strand break (DSB) relative biological effectiveness (RBE) for photons, neutrons, and light ions. Phys. Med. Biol..

[43] Stewart RD (2018). A comparison of mechanism-inspired models for particle relative biological effectiveness (RBE). Med. Phys..

