# Supplementary material for: Small-scale (sub-organ and cellular level) alpha-particle dosimetry methods using an iQID digital autoradiography imaging system
Source: Sci Rep. 2022 Oct 26;12:17934. doi: 10.1038/s41598-022-22664-5 (PMC9606121; doi:10.1038/s41598-022-22664-5)
Supplement: Supplementary file 1 — Supplementary Information. [file 41598_2022_22664_MOESM1_ESM.pdf]

Supplementary Information for

Small-Scale (Sub-Organ and Cellular Level) Alpha-Particle Dosimetry Methods using an iQID Digital Autoradiography Imaging System

R. Peter, B.M. Sandmaier, M.P. Dion, S.H.L. Frost, E.B. Santos, A. Kenoyer, D.K. Hamlin, D.S. Wilbur, R.D. Stewart, D.R. Fisher, K. Vetter, Y. Seo, and B.W. Miller

SUPPLEMENTAL TABLE S1. Dogs treated with ^{211}At-anti-CD45 radioimmunotherapy.

Canine ID	Subject Weight (kg)	Injected ^{211}At Activity (MBq/kg)	Antibody Dose (mAb mg/kg)	Specific Activity (MBq ^{211}At/ mg mAb)
H695	12.4	8.44	0.50	16.9
H700	13.0	23.2	0.75	30.9
H707	9.7	14.1	0.75	18.7
H714	10.1	14.5	0.50	29.0
H719	7.9	13.7	0.75	18.2
H741[a]	12.3	14.0	0.50	28.0
H751	9.5	12.9	0.50	25.8
H764	10.9	14.6	0.50	29.2
H765	9.6	11.4	0.50	22.7

[a]Biopsies from this study were discarded from analysis because torn and folded samples outnumbered usable samples.
